# Prognostic impact of blood urea nitrogen to albumin ratio on patients with sepsis: a retrospective cohort study

**DOI:** 10.1038/s41598-023-37127-8

**Published:** 2023-06-20

**Authors:** Yuhe Wang, Shan Gao, Lei Hong, Tingting Hou, Huihui Liu, Meng Li, Shu Yang, Yong Zhang

**Affiliations:** 1 Department of Pulmonary and Critical Care Medicine, First Affiliated Hospital of Bengbu Medical College, Bengbu, China; 2 Anhui Province Key Laboratory of Clinical and Preclinical Research in Respiratory Disease, Bengbu, China; 3 School of Health Management, Bengbu Medical College, Bengbu, China

**Keywords:** Prognostic markers, Risk factors

## Abstract

To investigate the ability of the ratio of blood urea nitrogen (BUN) to serum albumin ratio (BAR) in patients with sepsis in intensive care units (ICUs) to predict the prognosis of short-and long-term death. Data are from the Marketplace for Intensive Care Medical Information IV (MIMIC-IV v2.0) database for patients with sepsis as defined by SEPSIS-3. The primary outcome was 30-day mortality and the secondary outcome was 360-day mortality. Kaplan–Meier (KM) survival curves were plotted to describe differences in BAR mortality in different subgroups and area under the curve (AUC) analysis was performed to compare the predictive value of sequential organ failure assessment (SOFA), BAR, blood urea nitrogen (BUN) and albumin. Multivariate Cox regression models and subgroup analysis were used to determine the correlation between BAR and 30-day mortality and 360-day mortality. A total of 7656 eligible patients were enrolled in the study with a median BAR of 8.0 mg/g, including 3837 in the ≤ 8.0 group and 3819 in the BAR > 8.0 group, with 30-day mortality rates of 19.1% and 38.2% (*P* < 0.001) and 360-day mortality rates of 31.1% and 55.6% (*P* < 0.001). Multivariate Cox regression models showed an increased risk of death for 30-day mortality (HR = 1.219, 95% CI 1.095–1.357; *P* < 0.001) and 360-day mortality (HR = 1.263, 95% CI 1.159–1.376; *P* < 0.001) in the high BAR group compared to the low BAR group. For the 30-day outcome, the area under the curve (AUC) was 0.661 for BAR and 0.668 for 360-day BAR. In the subgroup analysis, BAR remained an isolated risk factor for patient death. As a clinically inexpensive and readily available parameter, BAR can be a valuable forecaster of prognosis in patients with sepsis in the intensive care unit.

## Introduction

Sepsis is a symptom of infection-related physiological, pathological and biochemical abnormalities^1^. Despite the decreasing trend in sepsis morbidity and mortality over the years, there are still 48.9 million cases of sepsis, of which 11 million were fatal in 2017^2^. Severe sepsis can cause acute multi-organ dysfunction^3,4^; surviving patients with sepsis often have long-term sequelae such as impaired immune function, cognitive function, and mental health, which affect the long-term health-related quality of life and survival^5,6^. Many biomarkers and multiple scoring systems have been used to predict the prognosis of patients with sepsis; however, these tools are either expensive or not readily available^7–9^.We aimed to explore convenient laboratory markers to predict the prognosis of patients with sepsis. Blood urea nitrogen (BUN) is the major product of protein metabolism in the human body and is mainly excreted by the kidneys. In the presence of excessive protein catabolism or reduced glomerular filtration, BUN levels rise, which is an essential parameter of the patient's renal status and protein catabolism metabolism^10^. Albumin is also one of the most commonly used assays in clinical laboratories and has a significant impact on many physiological mechanisms^11^. Increased microvascular permeability in inflammatory states alters the distribution of intra- and extravascular albumin, resulting in decreased serum albumin concentrations in many critically ill patients^12^. The impact of BUN and albumin on the prognosis of patients with sepsis has been demonstrated^13,14^. To our knowledge, the effectiveness of the predictive value of the urea-to-serum albumin ratio (BAR), calculated as the quotient of BUN and albumin, has not been investigated in patients with sepsis. For this reason, we first sought to determine the correlation between BAR and prognosis in septic patients in the intensive care unit (ICU).

## Materials and methods

### Data sources

Data were obtained from the Marketplace for Intensive Care Medical Information IV (MIMIC-IV version 2.0) database^15^, an open-source, free database developed by the affiliated laboratories at the Massachusetts Institute of Technology (MIT). The database records clinical data (e.g., patient baseline information, baseline vital signs, imaging tests, complications, medication use, and diagnoses) for patients admitted to a single-center intensive care unit from 2008 to 2019.MIMIC-IV is an updated clinical database that incorporates contemporary data and improves many aspects of MIMIC-III, a previously widely accepted database that has been intensively analyzed for academic purposes. This study has been granted access to the database by the relevant institution (certificate number: 39168475).

### Selection criteria

**Figure 1:**
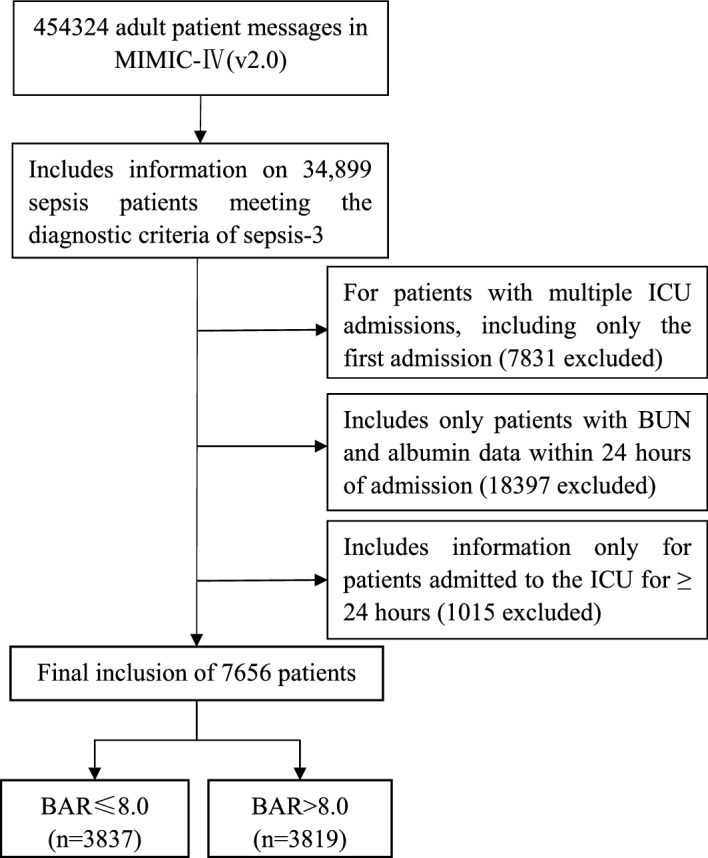
Flow Chart of the study.

According to the most recent definition of sepsis, SEPSIS-3, adult patients diagnosed with sepsis at the time of admission were included in this study. In addition, for patients re-entering the ICU, only patients admitted to the ICU for the first time were included in this study. We included the first BUN and albumin data for patients with sepsis within 24 h of ICU admission. Patients meeting the inclusion criteria were divided into two groups according to the median BAR (Fig. 1).

### Data collection

The following variables were extracted from the MIMIC-IV database. Patient demographic characteristics (age, gender), vital signs (heart rate, blood pressure, respiratory rate, oxygen saturation), laboratory parameters (red blood cells, white blood cells, platelets, hemoglobin, serum electrolytes, serum creatinine, glucose), and comorbidities (congestive heart failure, chronic pulmonary disease, liver disease, kidney disease, coronary arterial disease, malignant tumors, cerebrovascular disease, autoimmune disease, Peripheral vascular disease) at admission. Patients' comorbidities were extracted using the corresponding ICD codes. Extraction of data from the database using PostgreSQL structured query language, using a mock code base (https://github.com/MIT-LCP/mimic-code).

### Statistical analysis

Analysis of statistical data was performed using R, version 4.1.2 for Windows ("http://www.r-project.org/) and the Free Statistics analysis platform. All reported *P*-values are two-tailed, and P < 0.05 values were considered statistically significant. Continuous variables were presented as median ± interquartile difference; categorical variables were presented as frequencies; t-tests and X^2^ tests were used to compare differences between groups. KM survival curves were plotted for different subgroups according to BAR to show survival at 30 and 360 days in sepsis patients and compared using log-rank tests. Multivariable Cox regression models were used to estimate the association between BAR and all-cause mortality in sepsis. The multivariable Cox regression model was used to predict the interaction between BAR and all-cause mortality in sepsis. The results of the multivariable Cox regression models showed hazard ratios (HRs) and risk ratios with 95% confidence intervals (CIs). The model I was adjusted for age and sex. Model II was adjusted for age, heart rate, blood pressure, respiratory rate, oxygen saturation, comorbidities, erythrocytes, leukocytes, platelets, hemoglobin, serum potassium, serum creatinine, blood glucose, SOFA, and SAPS II. The predictive efficiency of BAR, SOFA, BUN, and albumin was compared using ROC curves. Subgroup analysis was used to assess the association between BAR and 30- and 360-day mortality, including age, sex, comorbidity, SOFA score, and SAPS II score.

### Outcomes

The main outcome measure was 30-day post-ICU mortality and the secondary outcome measure was 360-day post-ICU mortality.

## Results

### Population characteristics

A series of 7656 patients fulfilling the SEPSIS-3 diagnostic criteria with blood urea nitrogen and albumin data during 24 h in the ICU and completed follow-up were selected for this study, patients were divided into two groups by median, the data criteria selection process is shown in Fig. 1.

The baseline characteristics of the study population are shown in Table 1; patients in the high BAR group tended to be older, had a higher proportion of males, and had more comorbidities and adverse clinical signs, such as high respiratory rate, high white blood cell count; potassium, creatinine, glucose, and severity scores; and lower blood pressure, oxygen saturation, red blood cells, hemoglobin, platelets, and blood calcium levels compared to patients with low BAR (≤ 8.0). In addition, patients in the high BAR group had higher mortality rates at 30 and 360 days than those in the low groups.

**Table 1:** Characteristics of the study patients by BAR levels.

Characteristics	BAR levels			*P*
	Total (n = 7656)	BAR ≤ 8.0 (n = 3837)	BAR > 8.0 (n = 3819)	
Age (years), median (IQR)	64.0 (53.0, 76.0)	61.0 (49.0, 73.0)	68.0 (57.0, 78.0)	< 0.001
Gender, F, n (%)	3285 (42.9)	1761 (45.9)	1524 (39.9)	< 0.001
Basic vital signs, median (IQR)				
Heart rate (bpm)	88.2 (76.4, 100.4)	87.8 (76.4, 100.2)	88.6 (76.3, 100.8)	0.255
SBP(mmHg)	112.7 (103.8, 125.0)	115.1 (105.5, 127.8)	110.6 (102.4, 122.0)	< 0.001
DBP(mmHg)	61.4 (55.0, 68.8)	63.4 (57.1, 71.1)	59.0 (53.0, 66.0)	< 0.001
MBP(mmHg)	75.6 (69.5, 83.2)	77.9 (71.7, 85.7)	73.2 (67.6, 80.4)	< 0.001
Respiratory rate (bpm)	19.7 (17.2, 22.8)	19.3 (17.1, 22.3)	20.1 (17.4, 23.2)	< 0.001
SPO_2_ (%)	97.1 (95.7, 98.5)	97.3 (95.8, 98.6)	97.0 (95.5, 98.4)	< 0.001
Comorbidities, n (%)				
Coronary arterial disease	1778 (23.2)	777 (20.3)	1001 (26.2)	< 0.001
Congestive heart failure	2335 (30.5)	871 (22.7)	1464 (38.3)	< 0.001
Chronic pulmonary disease	1949 (25.5)	883 (23)	1066 (27.9)	< 0.001
Kidney disease	1770 (23.1)	339 (8.8)	1431 (37.5)	< 0.001
Liver disease	1046 (13.7)	401 (10.5)	645 (16.9)	< 0.001
Malignant tumors	1235 (16.1)	568 (14.8)	667 (17.5)	0.002
Cerebrovascular disease	1055 (13.8)	633 (16.5)	422 (11.1)	< 0.001
Peripheral vascular disease	817 (10.7)	326 (8.5)	491 (12.9)	< 0.001
Autoimmune disease	158 (2.1)	75 (2.0)	83 (2.2)	0.501
Laboratory parameters, median (IQR)				
Red blood cell (10^3^/μL)	3.4 (2.9, 4.0)	3.6 (3.1, 4.2)	3.3 (2.8, 3.8)	< 0.001
White blood cell (10^3^/μL)	11.7 (7.8, 16.8)	11.3 (7.7, 15.9)	12.1 (8.0, 17.7)	< 0.001
Platelet (10^3^/μL)	182.0 (118.0, 258.0)	195.0 (132.0, 264.0)	166.0 (105.5, 247.0)	< 0.001
Hemoglobin(mg/dL)	10.4 (8.8, 11.9)	11.0 (9.4, 12.6)	9.8 (8.4, 11.2)	< 0.001
Calcium (mEq/L)	8.1 (7.6, 8.7)	8.2 (7.6, 8.7)	8.1 (7.5, 8.7)	0.002
Potassium(mEq/L)	4.1 (3.7, 4.6)	4.0 (3.6, 4.4)	4.3 (3.8, 4.9)	< 0.001
Sodium (mEq/L)	139.0 (135.0, 142.0)	139.0 (136.0, 141.0)	138.0 (134.0, 142.0)	0.008
Creatinine (mg/dL)	1.1 (0.8, 1.9)	0.8 (0.6, 1.1)	1.7 (1.2, 2.9)	< 0.001
Glucose (mg/dL)	130.0 (104.0, 173.0)	127.0 (104.0, 165.0)	133.0 (104.0, 183.0)	< 0.001
Scoring systems, median (IQR)				
SOFA	7.0 (4.0, 10.0)	5.0 (3.0, 8.0)	9.0 (6.0, 12.0)	< 0.001
SAPSII	40.0 (31.0, 51.0)	35.0 (27.0, 43.0)	47.0 (38.0, 57.0)	< 0.001
30-day mortality	2191 (28.6)	731 (19.1)	1460 (38.2)	< 0.001
360-day mortality	3315 (43.3)	1192 (31.1)	2123 (55.6)	< 0.001

*SBP* systolic blood pressure, *DBP* diastolic blood pressure, *MAP* mean arterial pressure, *SOFA* sequential organ failure assessment, *SAPS II* simplified acute physiology score II.

### Relationship between BAR and patient mortality

**Figure 2:**
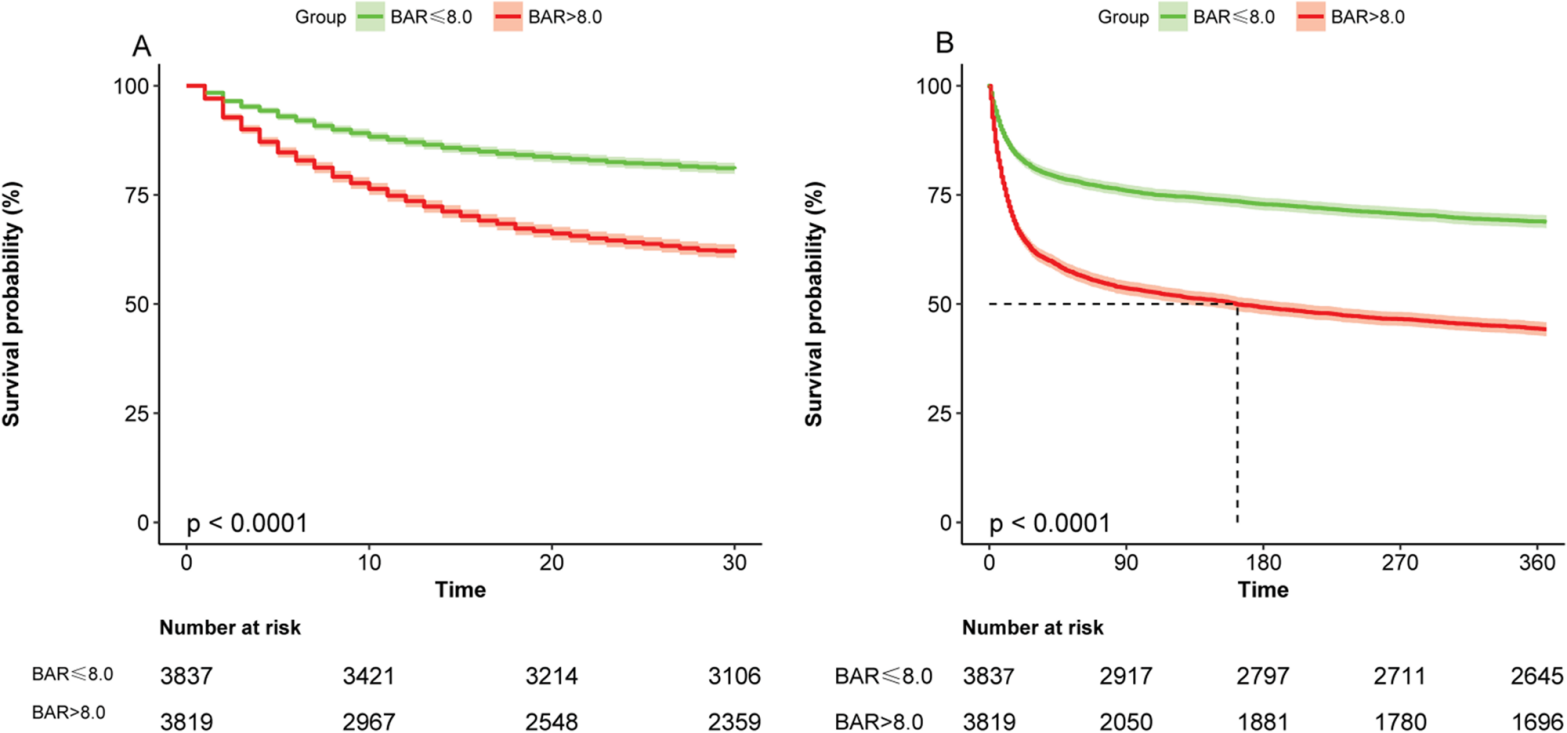
Kaplan–Meier curve presenting the relationship between BAR grouping and sepsis mortality. (**A**) Kaplan-Meier survival analysis curve for all-cause deaths within 30 days of ICU admission. (**B**) Kaplan-Meier survival analysis curve for all-cause deaths within 360 days of ICU admission.

Figure 2 shows the KM survival curves for the different groups; Fig. 2A shows the KM survival curve for 30-day death and Fig. 2B shows the KM survival curve for 360-day death. which were significantly lower in the high-BAR group than in the low-BAR group. The difference between the two curves was confirmed by the log-rank test (*P* < 0.001). In Table 2, multivariate Cox regression models showed that the high BAR group was associated with an increased risk of in-hospital mortality compared with the low BAR group.in 30-day mortality as the outcome (HR = 1.219, 95% CI 1.095–1.357, *P* < 0.001) and 360-day mortality (HR = 1.263, 95% CI 1.159–1.376, *P* < 0.001). Overall, higher BAR levels at ICU admission predicted higher mortality.

**Table 2:** HR (95% CIs) for all-cause mortality across groups of BAR level.

Factor	Univariate model		Model I		Model II	
	HR (95% CIs)	*P*	HR (95% CIs)	*P*	HR (95% CIs)	*P*
30-day all-cause mortality	1.032 (1.029 − 1.035)	< 0.001	1.031 (1.028 − 1.034)	< 0.001	1.008 (1.003 − 1.012)	< 0.001
BAR ≤ 8.0	Reference	–	Reference	–	Reference	–
BAR > 8.0	2.272 (2.079 − 2.483)	< 0.001	2.071 (1.892 − 2.267)	< 0.001	1.219 (1.095 − 1.357)	< 0.001
360-day all-cause mortality	1.032 (1.030 − 1.035)	< 0.001	1.030 (1.028 − 1.033)	< 0.001	1.011 (1.007 − 1.015)	< 0.001
BAR ≤ 8.0	Reference	–	Reference	–	Reference	–
BAR > 8.0	2.195 (2.045 − 2.357)	< 0.001	1.969 (1.832 − 2.116)	< 0.001	1.263 (1.159 − 1.376)	< 0.001

*HR* hazard ratio, *CIs* confidence intervals.

Model I covariates were adjusted for age and sex.

Model II covariates were adjusted for Age, systolic blood pressure, diastolic blood pressure, mean arterial pressure, respiration, oxygen saturation, congestive heart failure, kidney disease, malignancy, liver disease, peripheral vascular disease, Cerebrovascular Disease, red blood cells, white blood cells, platelets, hemoglobin, serum potassium, serum creatinine, blood glucose, SOFA, SAPS II.

### ROC curve analysis

**Figure 3:**
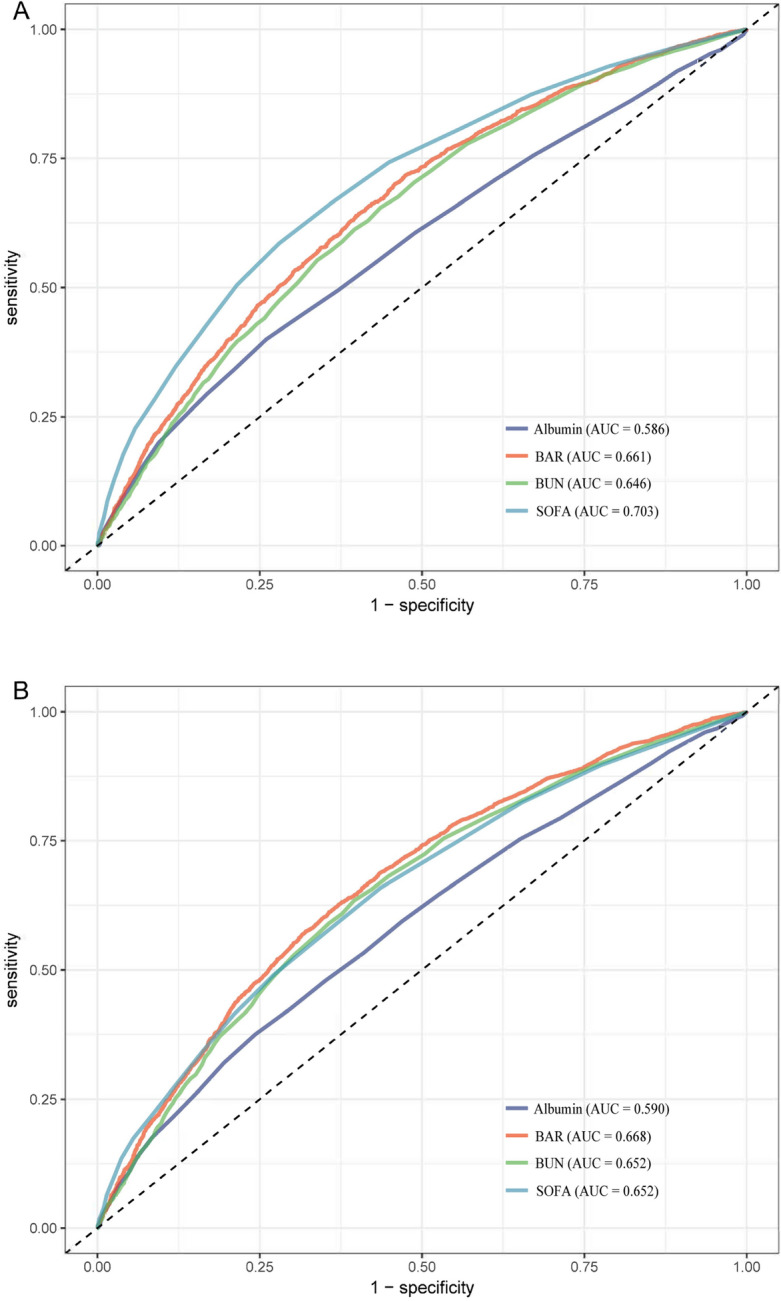
Receiver operating characteristic curve analysis for mortality in sepsis patients. (**A**) Receiver operating characteristic curve analysis for 30-day mortality in sepsis patients. (**B**) Receiver operating characteristic curve analysis for 360-day mortality in sepsis patients. *BUN* blood urea nitrogen, *BAR* blood urea nitrogen/serum albumin ratio, *SOFA* sequential organ failure assessment.

Figure 3A and B show the predictive value of SOFA, BAR, BUN, and albumin for 30-day mortality and 360-day mortality in patients with sepsis. The AUC of the receiver operating characteristic curve for 30-day mortality was 0.703 for SOFA, which had the best predictive value for 30-day patient outcome, and the AUC of 0.661 for BAR was slightly lower than for SOFA but better than for BUN and albumin alone. The AUC for BAR was 0.661, slightly lower than SOFA but better than BUN and albumin alone; the AUC for the receiver operating characteristic curve for 360-day mortality was 0.668, better than the other three for 360-day patient outcomes.

### Subgroup analysis

**Figure 4:**
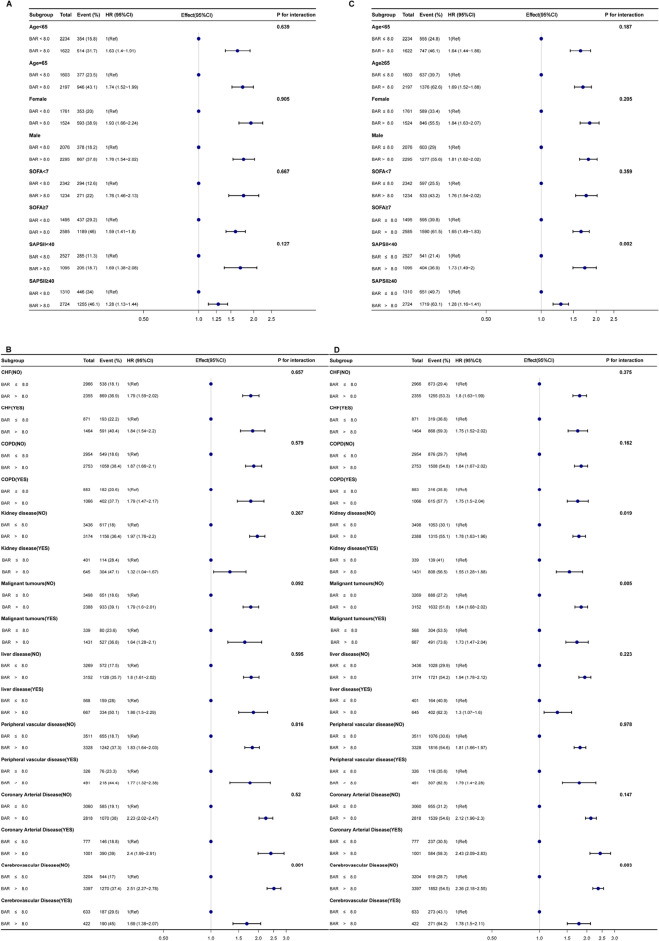
Forest plots for subgroup analysis of the relationship between mortality and BAR. (**A**, **B**) Forest plots for subgroup analysis of the relationship between 30-day mortality and BAR. (**C**, **D**) Forest plots for subgroup analysis of the relationship between 360-day mortality and BAR. *CHF* congestive heart failure, *COPD* chronic obstructive pulmonary disease.

To further investigate whether BAR remained an independent prognostic factor in specific subgroups of patients with sepsis, we performed an exploratory subgroup analysis of age, sex, comorbidity, and severity scores. Forest plots showed that BAR was an independent prognostic factor in most subgroups with 30-day mortality as the outcome, except for cerebrovascular disease (Fig. 4A and B). Similar results were found for 360-day mortality. Higher BAR still predicted higher mortality in all subgroups except for the interaction of patients with cerebrovascular disease, malignant tumors, and renal disease (Fig. 4C and D).

## Discussion

We retrospectively analyzed 7656 eligible patients from the MIMIC-IV (V2.0) database to examine the association between BAR and 30-day and 360-day mortality in patients with sepsis admitted to the ICU. Multivariate Cox regression and subgroup analysis showed that higher BAR values on admission were an independent risk factor and that high BAR values predicted higher mortality. We also compared the area under the curve for SOFA, BAR, BUN, and albumin using ROC curve analysis. For 30-day mortality, SOFA had the best predictive value and BAR had a better AUC than blood urea nitrogen and albumin, and for 360-day mortality, BAR had the best predictive value with a higher area under the curve than SOFA, BUN, and albumin. The area under the curve for BAR was also slightly higher for the 360-day mortality outcome than for the 30-day mortality outcome, suggesting that BAR is useful for predicting both long and short-term prognosis and has a better long-term predictive value for patients.

The pathophysiology of sepsis is a complex process of the host’s response to infection, localizing and controlling bacterial invasion while initiating the repair of damaged tissues. One of the mechanisms is the production of pro- and anti-inflammatory mediators^16^. Sepsis occurs when the release of pro-inflammatory mediators in response to infection is excessive or disproportionate (the so-called cytokine storm), leading to various organ dysfunctions^17,18^.

In sepsis, increased microvascular permeability in an inflammatory state alters the intra- and extravascular distribution of albumin, resulting in lower serum albumin concentrations in critically ill patients^19^. TNF-α and interleukin-1 can inhibit the transcription of albumin genes, thereby reducing serum albumin levels^20^, and Kendall et al., have confirmed that low serum albumin level is associated with poor prognosis in patients with sepsis^21^. As a well-known indicator of renal function, BUN may also reflect the complex relationship between a patient's nutritional status, protein metabolism, and renal status^10,22^. In patients with sepsis, RBF-independent microcirculatory dysfunction in the renal parenchyma is characterized by inflammatory mediators, immune cell infiltration, nitric oxide synthase dysregulation^23,24^, redistribution of blood flow from the renal medulla to the renal cortex, with some degree of renal medullary deoxygenation^25–27^, and is often complicated by acute kidney injury^28^. Critically ill patients are in a high proteolytic state^29^, and these factors can lead to elevated BUN levels in patients with sepsis. The effect of BUN on the prognosis of patients with sepsis has also been demonstrated.

Recently, BAR has been used as a new biomarker to assess the prognosis of various diseases and is an important prognostic factor for death in several diseases (lung cancer, gastrointestinal bleeding, community-acquired pneumonia, and others)^30–32^. Our study reported a strong correlation between BAR levels and 30-day and 360-day mortality in patients with sepsis, with a good predictive value.

The MIMIC-IV (v2.0) database, which will be released in June 2022, contains a large number of sepsis patients with complete follow-up data^28^ and provided us with data to support our study of the long-term prognosis of sepsis patients. The wealth of patient data in this database allowed us to perform ideal stratification and subgroup analyses. Despite the important findings we have highlighted, there were some limitations to this study. First, as with all retrospective analyses, there may be other confounding factors, and we adjusted for some common confounders to ensure the accuracy of our conclusions. Second, we excluded patients with multiple admissions with confirmed sepsis and only included patients with their first confirmed sepsis. This introduced the potential for selection bias. Finally, some patients were excluded from this study due to missing data, which may have biased the results of this study. A larger multi-center prospective study is needed for future validation.

## Conclusion

In summary, we found that high BAR was significantly associated with increased all-cause mortality in patients with sepsis and that BAR is a simple and effective biomarker in adults with sepsis.

## Supplementary Information

Supplementary Information.

## Data Availability

The datasets used and/or analyzed during the current study are available from the corresponding author upon reasonable request.
