# Supplementary material for: Prognostic impact of blood urea nitrogen to albumin ratio on patients with sepsis: a retrospective cohort study
Source: Sci Rep. 2023 Jun 20;13:10013. doi: 10.1038/s41598-023-37127-8 (PMC10282077; doi:10.1038/s41598-023-37127-8)
Supplement: Supplementary file 1 — Supplementary Information. [file 41598_2023_37127_MOESM1_ESM.docx]

Coronary artery disease codes:

SELECT * FROM "mimic_hosp"."diagnoses_icd " WHERE "icd_code" = 'I25' OR "icd_code" = 'I251' OR "icd_code" = 'I2510' OR "icd_code" = 'I2511' OR "icd_code" = 'I25110' OR "icd_code" = 'I25111' OR "icd_code" = 'I25118' OR "icd_code" = 'I25119' OR "icd_code" = '41400' OR "icd_code" = '41401' OR "icd_code" = '41402' OR "icd_code" = '41403' OR "icd_code" = '41404' OR "icd_code" = '41405' OR "icd_code" = '41406' OR "icd_code" = '41407'

Congestive heart failure codes:

max(

CASE

WHEN ((substr((diag.icd9_code)::text, 1, 3) = '428'::text) OR (substr((diag.icd9_code)::text, 1, 5) = ANY (ARRAY['39891'::text, '40201'::text, '40211'::text, '40291'::text, '40401'::text, '40403'::text, '40411'::text, '40413'::text, '40491'::text, '40493'::text])) OR ((substr((diag.icd9_code)::text, 1, 4) >= '4254'::text) AND (substr((diag.icd9_code)::text, 1, 4) <= '4259'::text)) OR (substr((diag.icd10_code)::text, 1, 3) = ANY (ARRAY['I43'::text, 'I50'::text])) OR (substr((diag.icd10_code)::text, 1, 4) = ANY (ARRAY['I099'::text, 'I110'::text, 'I130'::text, 'I132'::text, 'I255'::text, 'I420'::text, 'I425'::text, 'I426'::text, 'I427'::text, 'I428'::text, 'I429'::text, 'P290'::text]))) THEN 1

ELSE 0

END) AS congestive_heart_failure

Chronic pulmonary disease codes:

max(

CASE

WHEN ((substr((diag.icd9_code)::text, 1, 3) = ANY (ARRAY['440'::text, '441'::text])) OR (substr((diag.icd9_code)::text, 1, 4) = ANY (ARRAY['0930'::text, '4373'::text, '4471'::text, '5571'::text, '5579'::text, 'V434'::text])) OR ((substr((diag.icd9_code)::text, 1, 4) >= '4431'::text) AND (substr((diag.icd9_code)::text, 1, 4) <= '4439'::text)) OR (substr((diag.icd10_code)::text, 1, 3) = ANY (ARRAY['I70'::text, 'I71'::text])) OR (substr((diag.icd10_code)::text, 1, 4) = ANY (ARRAY['I731'::text, 'I738'::text, 'I739'::text, 'I771'::text, 'I790'::text, 'I792'::text, 'K551'::text, 'K558'::text, 'K559'::text, 'Z958'::text, 'Z959'::text]))) THEN 1

ELSE 0

END) AS peripheral_vascular_disease

Kidney disease codes:

max(

CASE

WHEN ((substr((diag.icd9_code)::text, 1, 3) = ANY (ARRAY['582'::text, '585'::text, '586'::text, 'V56'::text])) OR (substr((diag.icd9_code)::text, 1, 4) = ANY (ARRAY['5880'::text, 'V420'::text, 'V451'::text])) OR ((substr((diag.icd9_code)::text, 1, 4) >= '5830'::text) AND (substr((diag.icd9_code)::text, 1, 4) <= '5837'::text)) OR (substr((diag.icd9_code)::text, 1, 5) = ANY (ARRAY['40301'::text, '40311'::text, '40391'::text, '40402'::text, '40403'::text, '40412'::text, '40413'::text, '40492'::text, '40493'::text])) OR (substr((diag.icd10_code)::text, 1, 3) = ANY (ARRAY['N18'::text, 'N19'::text])) OR (substr((diag.icd10_code)::text, 1, 4) = ANY (ARRAY['I120'::text, 'I131'::text, 'N032'::text, 'N033'::text, 'N034'::text, 'N035'::text, 'N036'::text, 'N037'::text, 'N052'::text, 'N053'::text, 'N054'::text, 'N055'::text, 'N056'::text, 'N057'::text, 'N250'::text, 'Z490'::text, 'Z491'::text, 'Z492'::text, 'Z940'::text, 'Z992'::text]))) THEN 1

ELSE 0

END) AS kidney_disease

Liver disease codes:

max(

CASE

WHEN ((substr((diag.icd9_code)::text, 1, 4) = ANY (ARRAY['4560'::text, '4561'::text, '4562'::text])) OR ((substr((diag.icd9_code)::text, 1, 4) >= '5722'::text) AND (substr((diag.icd9_code)::text, 1, 4) <= '5728'::text)) OR (substr((diag.icd10_code)::text, 1, 4) = ANY (ARRAY['I850'::text, 'I859'::text, 'I864'::text, 'I982'::text, 'K704'::text, 'K711'::text, 'K721'::text, 'K729'::text, 'K765'::text, 'K766'::text, 'K767'::text]))) THEN 1

ELSE 0

END) AS liver_disease

Autoimmune disease codes:

SELECT * FROM "mimic_hosp"."diagnoses_icd" WHERE "icd_code" BETWEEN 'M30' AND 'M368' OR "icd_code" BETWEEN '4460' AND '4467' OR "icd_code" BETWEEN '7100' AND '7109'

Malignant tumors codes:

max(

CASE

WHEN (((substr((diag.icd9_code)::text, 1, 3) >= '140'::text) AND (substr((diag.icd9_code)::text, 1, 3) <= '172'::text)) OR ((substr((diag.icd9_code)::text, 1, 4) >= '1740'::text) AND (substr((diag.icd9_code)::text, 1, 4) <= '1958'::text)) OR ((substr((diag.icd9_code)::text, 1, 3) >= '200'::text) AND (substr((diag.icd9_code)::text, 1, 3) <= '208'::text)) OR (substr((diag.icd9_code)::text, 1, 4) = '2386'::text) OR (substr((diag.icd10_code)::text, 1, 3) = ANY (ARRAY['C43'::text, 'C88'::text])) OR ((substr((diag.icd10_code)::text, 1, 3) >= 'C00'::text) AND (substr((diag.icd10_code)::text, 1, 3) <= 'C26'::text)) OR ((substr((diag.icd10_code)::text, 1, 3) >= 'C30'::text) AND (substr((diag.icd10_code)::text, 1, 3) <= 'C34'::text)) OR ((substr((diag.icd10_code)::text, 1, 3) >= 'C37'::text) AND (substr((diag.icd10_code)::text, 1, 3) <= 'C41'::text)) OR ((substr((diag.icd10_code)::text, 1, 3) >= 'C45'::text) AND (substr((diag.icd10_code)::text, 1, 3) <= 'C58'::text)) OR ((substr((diag.icd10_code)::text, 1, 3) >= 'C60'::text) AND (substr((diag.icd10_code)::text, 1, 3) <= 'C76'::text)) OR ((substr((diag.icd10_code)::text, 1, 3) >= 'C81'::text) AND (substr((diag.icd10_code)::text, 1, 3) <= 'C85'::text)) OR ((substr((diag.icd10_code)::text, 1, 3) >= 'C90'::text) AND (substr((diag.icd10_code)::text, 1, 3) <= 'C97'::text))) THEN 1

ELSE 0

END) AS malignant_cancer

Peripheral vascular disease codes:

max(

CASE

WHEN ((substr((diag.icd9_code)::text, 1, 3) = ANY (ARRAY['440'::text, '441'::text])) OR (substr((diag.icd9_code)::text, 1, 4) = ANY (ARRAY['0930'::text, '4373'::text, '4471'::text, '5571'::text, '5579'::text, 'V434'::text])) OR ((substr((diag.icd9_code)::text, 1, 4) >= '4431'::text) AND (substr((diag.icd9_code)::text, 1, 4) <= '4439'::text)) OR (substr((diag.icd10_code)::text, 1, 3) = ANY (ARRAY['I70'::text, 'I71'::text])) OR (substr((diag.icd10_code)::text, 1, 4) = ANY (ARRAY['I731'::text, 'I738'::text, 'I739'::text, 'I771'::text, 'I790'::text, 'I792'::text, 'K551'::text, 'K558'::text, 'K559'::text, 'Z958'::text, 'Z959'::text]))) THEN 1

ELSE 0

END) AS peripheral_vascular_disease

Cerebrovascular disease codes:

max(

CASE

WHEN (((substr((diag.icd9_code)::text, 1, 3) >= '430'::text) AND (substr((diag.icd9_code)::text, 1, 3) <= '438'::text)) OR (substr((diag.icd9_code)::text, 1, 5) = '36234'::text) OR (substr((diag.icd10_code)::text, 1, 3) = ANY (ARRAY['G45'::text, 'G46'::text])) OR ((substr((diag.icd10_code)::text, 1, 3) >= 'I60'::text) AND (substr((diag.icd10_code)::text, 1, 3) <= 'I69'::text)) OR (substr((diag.icd10_code)::text, 1, 4) = 'H340'::text)) THEN 1

ELSE 0

END) AS cerebrovascular_disease,
